# Incidence, associated factors, and outcomes of acute kidney injury following placement of antibiotic bone cement spacers in two-stage exchange for periprosthetic joint infection: a comprehensive study

**DOI:** 10.3389/fcimb.2023.1243290

**Published:** 2023-09-20

**Authors:** Zhuo Li, Zulipikaer Maimaiti, Fan Yang, Jun Fu, Zhi-Yuan Li, Li-Bo Hao, Ji-Ying Chen, Chi Xu

**Affiliations:** ^1^School of Medicine, Nankai University, Tianjin, China; ^2^Department of Orthopedics, The First Medical Center, Chinese PLA General Hospital, Beijing, China; ^3^Department of Orthopedics, The Fourth Medical Center, Chinese PLA General Hospital, Beijing, China

**Keywords:** periprosthetic joint infection, antibiotic bone cement spacer, acute kidney injury, incidence, associated factors, outcome

## Abstract

**Background:**

Two-stage exchange with placement of antibiotic cement spacer (ACS) is the gold standard for the treatment of chronic periprosthetic joint infection (PJI), but it could cause a high prevalence of acute kidney injury (AKI). However, the results of the current evidence on this topic are too mixed to effectively guide clinical practice.

**Methods:**

We retrospectively identified 340 chronic PJI patients who underwent the first-stage exchange with placement of ACS. The Kidney Disease Improving Global Outcomes guideline was used to define postoperative AKI. Multivariate logistic analysis was performed to determine the potential factors associated with AKI. Furthermore, a systematic review and meta-analysis on this topic were conducted to summarize the knowledge in the current literature further.

**Results:**

In our cohort, the incidence of AKI following first-stage exchange was 12.1%. Older age (per 10 years, OR= 1.509) and preoperative hypoalbuminemia (OR= 3.593) were independent predictors for postoperative AKI. Eight AKI patients progressed to chronic kidney disease after 90 days. A meta-analysis including a total of 2525 PJI patients showed the incidence of AKI was 16.6%, and AKI requiring acute dialysis was 1.4%. Besides, host characteristics, poor baseline liver function, factors contributing to acute renal blood flow injury, and the use of nephrotoxic drugs may be associated with the development of AKI. However, only a few studies supported an association between antibiotic dose and AKI.

**Conclusion:**

AKI occurs in approximately one out of every six PJI patients undergoing first-stage exchange. The pathogenesis of AKI is multifactorial, with hypoalbuminemia could be an overlooked associated factor. Although the need for acute dialysis is uncommon, the fact that some AKI patients will develop CKD still needs to be taken into consideration.

## 1 Introduction

Periprosthetic joint infection (PJI) is a catastrophic complication after total joint arthroplasty (TJA), with incidence rates ranging from 0.5% to 2% (Edwards et al., 2009; Namba et al., 2013). It takes a tremendous toll on patients’ physical and mental health, often putting them at a higher risk of death, and adding a heavy financial burden to the healthcare system (Kurtz et al., 2008; Zmistowski et al., 2013; Premkumar et al., 2021). A two-stage exchange involving the placement of a high-dose antibiotic-loaded cement spacer (ACS) supplemented with intravenous or oral pathogen-sensitive antibiotics is the standard approach for treating chronic PJI (Cha et al., 2015; Charette and Melnic, 2018). This technique has proven effective, with studies reporting reliable eradication of infection and long-term prevention of reinfection (Haleem et al., 2004; Engesaeter et al., 2011; Cooper and Della Valle, 2013; Puhto et al., 2014).

However, the most commonly used antibiotics in two-stage exchange, such as aminoglycosides and vancomycin, are highly nephrotoxic (Humes, 1988; Rybak et al., 1990). The systemic absorption of high-dose antibiotics in ACS, especially when combined with intravenous antibiotics, anesthetic drugs, and surgical procedures, could increase the risk of acute kidney injury (AKI). Given that AKI is associated with an acute demand for dialysis, prolonged hospital stays, and increased mortality, there is an urgent need to deepen clinicians’ understanding of AKI (Luu et al., 2013; Theil et al., 2021; Valenzuela et al., 2022).

The incidence of AKI following placement of ACS in two-stage exchange varies considerably in the literature (Geller et al., 2017; Berliner et al., 2018; Edelstein et al., 2018; Yadav et al., 2018; Theil et al., 2021; Dagneaux et al., 2021a; Dagneaux et al., 2021b; Valenzuela et al., 2022), ranging from 0% to 33.3% (Springer et al., 2004; Hsieh et al., 2006; Gooding et al., 2011; Theil et al., 2021). Luu et al. (2013) performed a preliminary systematic review of 544 patients, showing an AKI incidence of 4.8%. However, their major limitation was that AKI was not the primary endpoint in most included studies, and the definition of AKI was unclear. These may result in a significant underestimation of the incidence of AKI, even lower than the incidence of approximately 6.3% following primary TJA (Thongprayoon et al., 2019). In addition, a North American study reported that AKI developed in 3.4% of 2147 patients for aseptic reasons, and the incidence of AKI may be significantly higher in PJI patients (Yadav et al., 2018). Furthermore, although some recent studies attempted to explore potential risk factors for AKI following first-stage exchange, they often provided fragmented and conflicting knowledge (Menge et al., 2012; Geller et al., 2017; Berliner et al., 2018; Yadav et al., 2018; Theil et al., 2021; Valenzuela et al., 2022). Information regarding renal outcomes after AKI is also very limited.

Overall, the results of the current evidence on this topic were too mixed to effectively guide clinical practice. We therefore conducted a retrospective institutional study and a systematic literature review to summarize the evidence on the incidence, associated factors, and outcomes of AKI after placement of ACS in two-stage exchange.

## 2 Materials and methods

### 2.1 Study population

**Figure 1:**
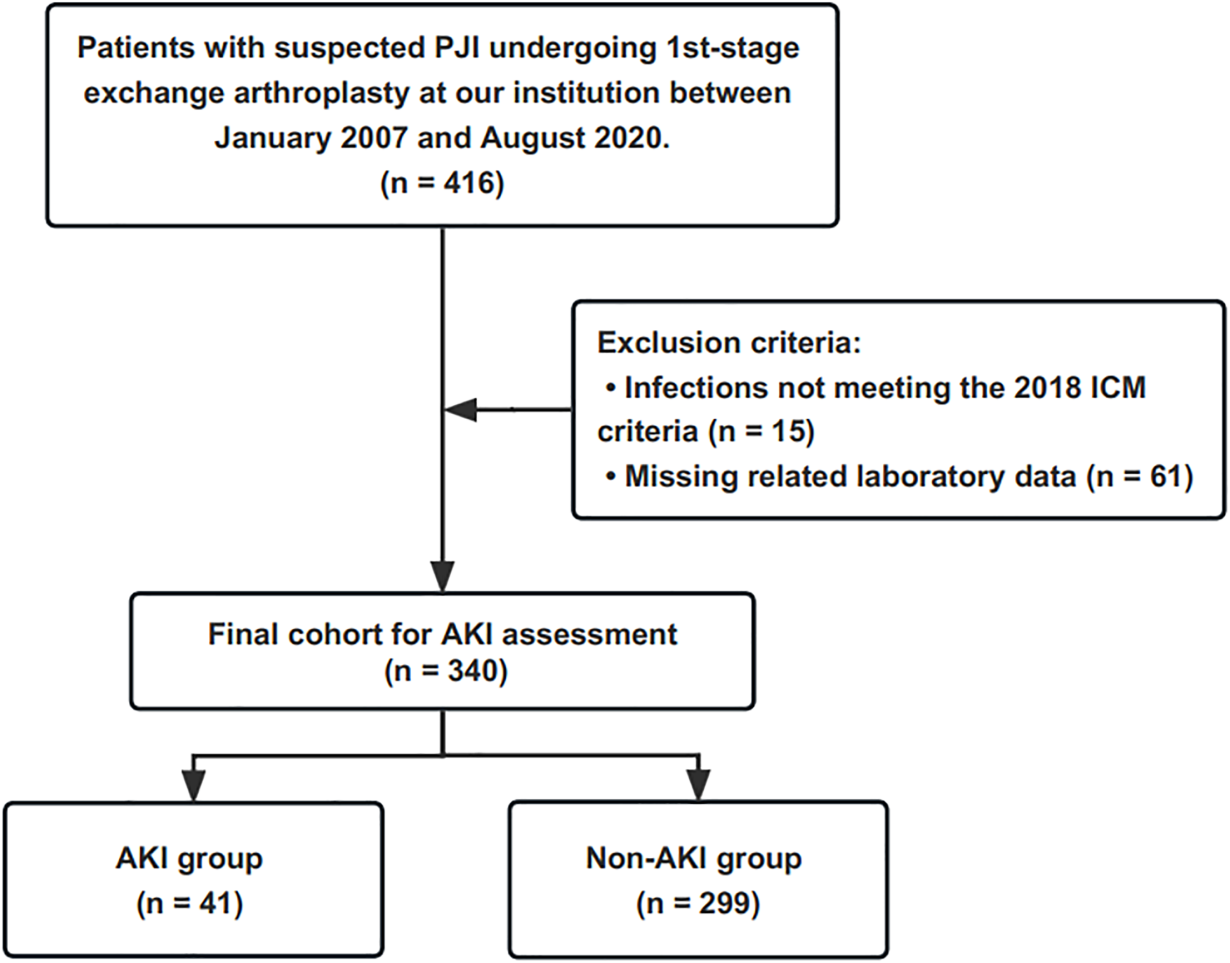
This flowchart demonstrates how patients are identified for inclusion in the final cohort. PJI, periprosthetic joint infection; ICM, International Consensus Meeting; AKI, acute kidney injury.

The retrospective study was conducted at a national high-volume PJI treatment center. After the Institutional Review Board’s approval, we retrospectively reviewed our institution’s two-stage revision database. Four hundred and sixteen patients with suspected PJI underwent the first-stage resection between January 2007 and August 2020 were identified. We excluded 15 patients who did not meet the International Consensus Meeting (ICM) 2018 criteria (Shohat et al., 2019), and sixty-one patients with missing laboratory test data. Three hundred and forty patients were included in the final cohort, and the flow chart for the enrolment of PJI patients is shown in **Figure 1**.

### 2.2 Two-stage exchange technique

Institutional-based surgical approaches performed by high-volume surgeons were applied to all patients. The first-stage resection consists primarily of removing the infected prosthesis, thorough debridement, and placement of an articulating or static ACS. An ACS was made by intraoperatively mixing bone cement (pre-mixed with gentamicin) with additional antibiotic powder (e.g. vancomycin). As a general rule, 4g of antibiotic powder was impregnated in every 40g of bone cement. Postoperative pathogen-sensitive antibiotics were administered systemically based on cultures from joint aspirations. If the cultures were negative, broad-spectrum antibiotics such as vancomycin would be administered. After retaining the spacer for at least eight weeks, patients underwent a second-stage reimplantation if they were assessed as clear of infection.

### 2.3 Clinical data extraction

Two investigators independently reviewed the patient’s medical records, surgical notes, and medical orders to extract clinical information associated with the development of AKI. Variables included: age, sex, Body Mass Index (BMI), type of antibiotics (administered for at least three days), comorbidities, type of pathogen, and laboratory values of blood urea nitrogen (BUN), blood uric acid (BUA), serum albumin, C-reactive protein (CRP), erythrocyte sedimentation rate (ESR), serum creatinine (SCr) and hemoglobin. The most recent preoperative SCr value was defined as the baseline. According to the care routine of our institution, patients were monitored for SCr levels on postoperative days 1, 3, 5, and 7, and these SCr values were extracted to assess the onset of AKI.

The baseline characteristics of the 340 PJI patients in the final cohort are summarized in **Table 1**. The patients were (60.1 ± 13.2) years old, and 50.0% were men with a mean BMI of (25.2 ± 3.8) kg/m2. The most common primary diagnosis was osteoarthritis (115/340, 33.8%).

**Table 1:** Baseline Demographic Details.

Characteristics	Total	AKI Group (N=41)	Non-AKI (N=299)	P
Age, years	60.1 ± 13.2	66.3 ± 10.1	59.2 ± 13.4	**0.001**
*Sex*				0.868
Man	170 (50.0)	20 (48.8)	150 (50.2)	
Woman	170 (50.0)	21 (51.2)	149 (49.8)	
*Joint*				0.854
Hip	187 (55)	22 (52.7)	165 (55.2)	
Knee	153 (45)	19 (47.2)	134 (44.8)	
BMI, kg/m^2^	25.2 ± 3.8	25.4 ± 3.8	25.1 ± 3.7	0.663
Asian race	340 (100)	41 (100)	299 (100)	–
Smoking	30 (8.8)	3 (7.3)	27 (9.0)	0.717
*Primary Diagnosis*				0.195
Osteoarthritis	115 (33.8)	18 (43.9)	97 (32.4)	
ONFH	85 (25.0)	10 (24.4)	75 (25.1)	
DDH	5 (1.5)	2 (4.9)	3 (1.0)	
Inflammatory arthritis	20 (5.9)	1 (2.4)	19 (6.4)	
Fracture	74 (21.8)	7 (17.1)	67 (22.4)	
Others	41 (12.1)	3 (7.3)	38 (12.7)	
Comorbidities				
Cardiovascular Disease	42 (12.4)	7 (17.1)	35 (11.7)	0.327
Kidney Disease	11 (3.2)	3 (7.3)	8 (2.7)	0.456
Diabetes	45 (13.2)	9 (22.0)	36 (12.0)	0.079
Number of Prior Surgery	1.6 ± 1.0	1.5 ± 0.8	1.6 ± 1.1	0.298
Presence of a Sinus Tract	132 (38.8)	17 (41.5)	115 (38.5)	0.711
History of Infected Surgery	191 (56.2)	21 (51.2)	170 (56.9)	0.495
Preoperative biomarkers				
Serum Creatinine, umol/L	63.2 ± 16.2	61.7 ± 15.1	63.4 ± 16.4	0.530
Hemoglobin, g/L	123.0 ± 15.9	121.6 ± 13.6	123.2 ± 16.2	0.560
Serum Albumin, g/L	38.5 ± 4.1	36.6 ± 3.8	38.7 ± 4.1	**0.002**
BUN, mmol/L	5.1 ± 1.7	5.1 ± 1.9	5.1 ± 1.7	0.857
BUA, μmol/L	301.7 ± 86.3	282.4 ± 81.8	304.4 ± 86.7	0.127
C-reactive Protein, mg/dL	2.4 ± 2.5	2.7 ± 2.1	2.4 ± 2.6	0.386
ESR, mm/h	42.6 ± 26.3	51.2 ± 27.0	41.4 ± 26.1	**0.025**

The numbers in brackets are standard deviations for continuous variables and percentages for categorical variables. AKI, acute kidney injury; BMI, body mass index; ONFH, osteonecrosis of the femoral head; DDH, developmental dysplasia of the hip; BUN, blood urea nitrogen; BUA, blood uric acid; ESR, erythrocyte sedimentation rate.P values with statistical significance are highlighted in bold. "-" means not applicable.

### 2.4 Study outcomes

The diagnosis of AKI was based on the Kidney Disease Improving Global Outcomes (KDIGO) clinical practice guideline. It was proposed in 2012 and was a refinement of the Risk, Injury, Failure, Loss, and End-stage renal disease (RIFLE) criteria and the Acute Kidney Injury Network (AKIN) criteria (Khwaja, 2012). Due to the lack of information on urine output, only SCr levels were used to evaluate AKI. Briefly, an increase in SCr level of 0.3 mg/dL within 48 hours after surgery, or an increase in SCr level of more than 1.5 times the patient’s preoperative baseline SCr level within one week, was diagnosed as AKI. Moreover, we further investigated whether AKI patients progressed to chronic kidney disease (CKD) (≥90 days).

### 2.5 Systematic review and meta-analysis

Moreover, a systematic literature search of the EMBASE, Pubmed, and Cochrane Database of Systematic Reviews was conducted in October 2022 to assess AKI’s incidence and relevant factors following the first-stage exchange. The keywords included terms related to “acute kidney injury,” “acute renal failure,” “periprosthetic joint infection,” “acute dialysis,” “two-stage exchange,” and “spacer,” with specific search strategies shown in **Supplementary Table 1**. The above eligibility criteria covered all original reports dealing with two-stage exchange arthroplasty and postoperative AKI, and they all reported a definition of diagnosed AKI. We further considered observational studies, clinical trials, and case series with AKI following the first-stage exchange as the primary outcome and for which incidence or risk factors were reported. Three independent researchers reviewed the titles and abstracts of the publications to determine their suitability. Additionally, references in the key publications were reviewed to supplement the document collection further. A structured spreadsheet was created to collect the following information: title, first author’s name, study year, country, study design, sample size, antibiotic information, the definition of AKI, incidence of AKI, and associated factors for AKI.

### 2.6 Statistical analysis

The study cohort was divided into AKI and non-AKI groups, and descriptive statistics were performed. Continuous variables were presented as mean ± standard deviation (SD) and compared using the t-test. Categorical data were presented as frequencies and percentages and compared using chi-square or Fisher’s exact test. We first performed a univariate logistic regression analysis for all variables (continuous variables are converted to categorical variables via receiver operating characteristic curves) to assess the relationship between baseline patient characteristics and AKI. Variables with *P* values <0.1 were further included in multifactorial logistic regression analysis to identify independent predictors of AKI. All logistic regression analyses reported adjusted odds ratios (ORs) and 95% confidence intervals (CIs). The above statistical analyses were performed by SPSS 25.0. *P*<0.05 was considered to indicate statistical significance.

Comprehensive Meta-Analysis V3 software was used to perform the meta-analysis. Adjusted point estimates were consolidated using a generic inverse variance method of DerSimonian and Laird (1986). The random-effects model was used to pool AKI incidence. The *I^2^* statistic was adopted to assess the heterogeneity of included studies, with less than 50% of *I^2^* considered low heterogeneity, 51% to 75% moderate heterogeneity, and greater than 76% high heterogeneity. The publication bias was evaluated by funnel plot and Egger test.

## 3 Results

### 3.1 Incidence of AKI following the first-stage exchange

In our study cohort, 41 patients (12.1%) developed AKI within the first seven days postoperatively. Compared to the non-AKI group, patients in the AKI group were significantly older (**Table 1**, 66.3 ± 10.1 vs. 59.2 ± 13.4, *P*= 0.01), had significantly lower serum albumin levels (36.6 ± 3.8 g/L vs. 38.7 ± 4.1 g/L, *P*= 0.002), and higher ESR levels (51.2 ± 27.0 mm/h vs. 41.4 ± 26.1 mm/h, *P*= 0.025). There were no significant differences between the two groups in terms of sex, BMI, joint, primary diagnosis, the presence of a sinus tract, number of prior open surgery, comorbidities, preoperative hemoglobin, BUN, BUA, SCr, and CRP levels. We also did not observe differences between the two groups regarding pathogenic organisms, with *coagulase-negative staphylococcus* being the most common (**Table 2**). Besides, the two groups had no differences in the type of spacer-loaded or systemically administered antibiotics (**Table 3**). Patients in the AKI group had higher SCr during the first week postoperatively than those in the non-AKI group (**Table 4**, *P*< 0.001). The highest SCr level was seen on the third postoperative day.

**Table 2:** Pathogen Data.

Identified Microorganisms	AKI Group (N=41)	Non-AKI (N=299)	*P*
*Staphylococcus aureus*	6 (14.6)	40 (13.4)	0.825
*Coagulase-negative Staphylococcus*	15 (36.6)	127 (42.5)	0.473
*Streptococcus*	0 (0)	17 (5.7)	0.117
*Entoococcus*	3 (7.3)	16 (5.4)	0.607
*Gram-Negative Bacteria*	2 (4.9)	28 (9.4)	0.342
*Fungi*	1 (2.4)	12 (4.0)	0.622
Others	2 (4.9)	20 (6.7)	0.658
Mixed	3 (7.3)	46 (15.4)	0.168
Negative	14 (34.1)	79 (26.4)	0.298

Numbers in brackets are percentages. AKI, acute kidney injury.

**Table 3:** Antibiotics Administered.

Systemic (Intravenous or Oral) Antibiotics	AKI Group (N=41)	Non-AKI (N=299)	*P*
Vancomycin	10 (24.4)	49 (16.4)	0.205
Quinolones	13 (31.7)	91 (30.4)	0.868
Linezolid	5 (12.2)	55 (18.4)	0.329
Rifapentine	6 (14.6)	46 (15.4)	0.900
Third-Generation Cephalosporin	1 (2.4)	20 (6.7)	0.289
Meropenem	0 (0)	8 (2.7)	0.289
Fluconazole/Voriconazole	1 (2.4)	12 (4.0)	0.622
Others	5 (12.2)	20 (6.7)	0.341
Antibiotics Added in Spacer			
Vancomycin	36 (87.8)	279 (93.3)	0.205
Meropenem/Imipenem	14 (34.1)	143 (47.8)	0.099
Others	8 (19.5)	51 (17.1)	0.841

Numbers in brackets are percentages. AKI, acute kidney injury.

**Table 4:** Trends of Creatinine in the First Seven Days Postoperatively.

SCr, umol/L	Total	AKI	Non-AKI	*P*
**Baseline**	63.2	61.7	63.4	0.530
**POD1**	65.0	83.8	62.4	<0.001
**POD3**	68.5	90.6	65.3	<0.001
**POD5**	66.0	85.7	63.3	<0.001
**POD7**	67.0	88.9	64.1	<0.001

SCr, Serum creatinine; AKI, acute kidney injury, POD, postoperative day.

### 3.2 Associated factors of AKI

As shown in **Table 5**, the univariate logistic regression analysis identified older age, diabetes, ESR > 40 mm/h, and hypoalbuminemia as potential predictors. Older age (per additional 10 years, OR= 1.509; 95%CI, 1.072-2.119; *P*= 0.019) and hypoalbuminemia (OR= 3.593; 95%CI, 1.688-7.650; *P*= 0.001) remained significant predictors in the multifactorial logistic model.

**Table 5:** Logistic Regression Model for Acute Kidney Injury.

Variables	Univariate		Multivariate	
	OR (95% CI)	*P*	OR (95% CI)	*P*
Older Age (per 10 years)	1.692 (1.219-2.367)	0.002	1.509 (1.072-2.119)	0.019
Diabetes	2.055 (0.907-4.653)	0.084	1.541 (0.646-3.673)	0.329
ESR > 40 mm/h	2.163 (1.101-4.250)	0.025	1.375 (0.662-2.856)	0.394
Hypoalbuminemia	4.843 (2.380-9.858)	<0.001	3.593 (1.688-7.650)	0.001

ESR, erythrocyte sedimentation rate; OR, odds ratio; CI, confidence interval.

### 3.3 Renal outcomes

No patient had acute dialysis needs during hospitalization. Nine AKI patients were diagnosed with CKD after 90 days, eight of whom did not suffer from pre-existing renal disease. Five patients with CKD were applied renal preservation therapy for more than one month and none of them were readmitted for CKD.

### 3.4 Systematic review and meta-analysis

**Figure 2:**
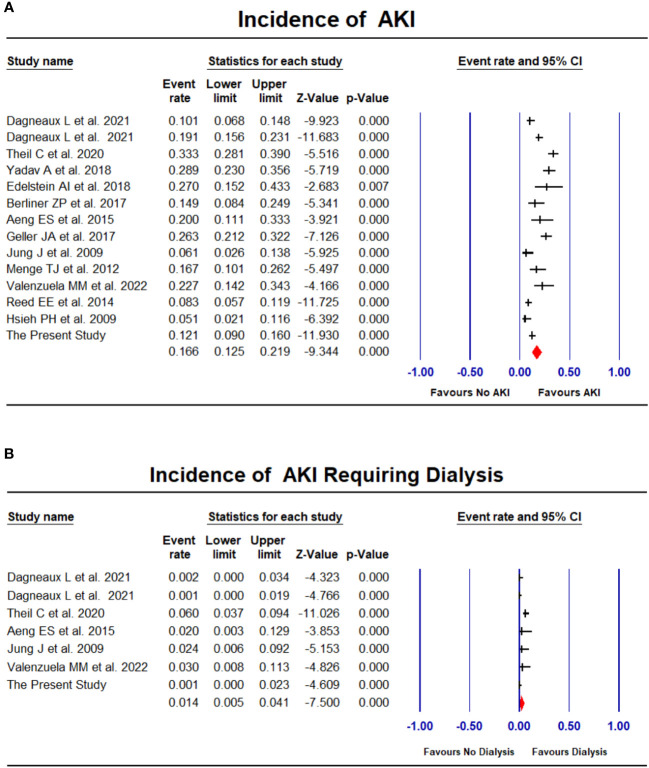
Forest plots of the included studies assessing incidence of AKI **(A)** and AKI requiring dialysis **(B)**.

Apart from our study, 13 studies (Hsieh et al., 2009; Jung et al., 2009; Menge et al., 2012; Reed et al., 2014; Aeng et al., 2015; Geller et al., 2017; Berliner et al., 2018; Edelstein et al., 2018; Yadav et al., 2018; Theil et al., 2021; Dagneaux et al., 2021a; Dagneaux et al., 2021b; Valenzuela et al., 2022) comprising additional 2185 PJI patients undergoing first-stage exchange were included in the meta-analysis of associated AKI incidence (**Table 6**). The pooled estimated incidence of AKI was 16.6% (95% CI: 12.5%-21.9%, *I*^2 ^= 79%, **Figure 2A**) and the incidence of AKI requiring dialysis was 1.4% (95% CI: 0.5%-4.1%, *I*^2 ^= 70%, **Figure 2B**). There was no significant publication bias in the meta-analysis assessing the incidence of AKI (Funnel plot was shown in **Supplementary Figure 1**, *P* for Egger test = 0.08).

**Table 6:** Systematic Review of AKI Following the First-Stage Exchange.

Study	Country	Design	Number of Patients (Male%)	Average Age (Years)	Spacer Retained	Systemic Antibiotics Used	AKI Definition	Incidence	Related Factors
Dagneaux et al., 2021a	USA	Cohort	227 (55)	65	15 weeks	Vancomycin (42%) cefazolin 51 (19.9%) ceftriaxone (16.8%)	KDIGO guidelines	10.1%	Postoperative fluid depletion and/or hypovolemia, acute atrial fibrillation, and CKD
Dagneaux et al., 2021a	USA	Cohort	424 (53)	67	11 weeks	Vancomycin (40%) cefazolin 51 (23%) ceftriaxone (13%)	KDIGO guidelines	19.1%	Hypertension, perioperative hypovolemia, CKD, acute atrial fibrillation, and higher concentrations of vancomycin or aminoglycosides in ACS
Theil et al., 2021	Germany	Cohort	285 (48)	–	13 weeks	Vancomycin, aminopenicillins, and linezolid	KDIGO guidelines	33.3%	Age and baseline SCr
Yadav et al., 2018	USA	Cohort	197 (54)	66	–	Cefazolin and vancomycin	RIFLE criteria	28.9%	Age and CCI
Edelstein et al., 2018	USA	Prospective cohort	37 (60)	67	8 weeks	Culture-directed intravenous antibiotics	RIFLE criteria	27.0%	–
Berliner ZP et al., 2017	USA	Cohort	74 (53)	67	–	–	A greater than 50% rise in SCr to a value of at least 1.4 mg/dL	14.9%	Lower baseline hemoglobin
Aeng et al., 2015	Canada	Prospective cohort	50 (54)	66	–	Cephalosporins, vancomycin, and rifampin	A greater than 50% rise in SCr within the first 7 days	20.0%	ACS premanufactured with gentamicin, administration of blood transfusions and NSAIDs postoperatively
Geller et al., 2017	USA	Cohort	247 (48)	64	–	Vancomycin (45%), daptomycin (21%), cefazolin (18%)	KDIGO guidelines	26.3%	Higher BMI, lower baseline hemoglobin level, and existence of a comorbid condition
Jung et al., 2009	Germany	Cohort	82 (52)	70	13 weeks	Primarily vancomycin and rifampicin	A greater than 50% rise in SCr	6.1%	–
Menge et al., 2012	USA	Cohort	84 (45)	63	–	–	A greater than 50% rise in SCr to a value of at least 1.4 mg/dL	16.7%	Dose of vancomycin or tobramycin in the ACS
Valenzuela et al., 2022	USA	RCT	66 (54)	68	–	Culture-targeted IV antibiotics	KDIGO guidelines	22.7%	Preoperative CKD
Reed et al., 2014	USA	Cohort	313	–	–	Primarily vancomycin, piperacillin/tazobactam	A SCr increase of 0.5 mg/dL or 50%	8.3%	ACE inhibitor exposure; piperacillin-tazobactam exposure
Hsieh et al., 2009	China	Cohort	99 (61)	–	–	1st-gen cephalosporin and gentamicin, else based on cultures and sensitivities	A SCr increase of 0.5 mg/dL or 50%	5.1%	–

AKI, acute kidney injury; SCr, serum creatinine; BMI, body mass index; ACS, antibiotic-loaded cement spacer; CKD, chronic kidney disease; CCI, Charlson comorbidity index; KDIGO, Kidney Disease: Improving Global Outcomes; RIFLE, risk, injury, failure, loss, end stage kidney disease; RCT, randomized controlled trial; ACE, angiotensin converting enzyme; NSAIDs, nonsteroidal anti-inflammatory drugs."-" means not applicable.

Reported related factors for AKI in PJI patients undergoing first-stage exchange are demonstrated in **Table 6**. The identified factors include demographic characteristics such as age (Yadav et al., 2018; Theil et al., 2021) and BMI (Geller et al., 2017); factors affecting renal perfusions such as hypovolemia (Dagneaux et al., 2021a; Dagneaux et al., 2021b), acute atrial fibrillation (Rybak et al., 1990; Haleem et al., 2004) and low baseline hemoglobin levels (Aeng et al., 2015; Berliner et al., 2018; Theil et al., 2021); poorer baseline renal function such as a history of chronic renal disease (Dagneaux et al., 2021a; Dagneaux et al., 2021b; Valenzuela et al., 2022) and higher creatinine levels (Aeng et al., 2015); and the use of specific medications such as nonsteroidal anti-inflammatory drugs (NSAIDs) (Edwards et al., 2009) and angiotensin-converting enzyme inhibitors (ACEIs) (Reed et al., 2014). A few studies have also reported on the potential impact of the type or dose of antibiotics (Menge et al., 2012; Reed et al., 2014; Dagneaux et al., 2021a).

## 4 Discussion

Despite the widespread use of two-stage exchange arthroplasty in the management of chronic PJI, data on acute kidney injury (AKI) following placement of antibiotic-loaded ACS are limited and the results are mixed. Our results indicated the incidence of AKI following first-stage exchange was 12.1% in our large retrospective cohort; a meta-analysis including total 2525 PJI patients demonstrated an AKI incidence of 16.6%. In addition to the identified demographic characteristics, factors contributing to acute renal blood flow impairment, and the use of several medications, hypoalbuminemia may be a new potential factor associated with the development of AKI. Although the need for acute dialysis is uncommon when AKI occurs, approximately 20% of AKI patients still progress to CKD.

### 4.1 Incidence of AKI

The incidence of AKI following first-stage revision reported in the literature varies substantially at present, which may be explained by differences in the definition of AKI and the spacer technique (Luu et al., 2013; Dagneaux et al., 2021a; Dagneaux et al., 2021b). Our meta-analysis showed an incidence of 16.6% for AKI defined by standard criteria, which is three to four times higher than those following primary total joint arthroplasty (Thongprayoon et al., 2019; Yayac et al., 2021). This incidence is of greater concern to clinicians and is higher than the incidence of AKI after major abdominal surgery (approximately 13.4% (O'Connor et al., 2016)). A slightly lower incidence of AKI was observed in our cohort, which may be due to the fact that the patients were younger. Meanwhile, the incidence of AKI varied across races and our study is the first to report the incidence of AKI in Asians in a large PJI cohort. Reports from other areas have shown that black patients tend to have a higher risk of AKI than white patients, and Asians also have a relatively low incidence of AKI (Hassan and Balogun, 2022). However, black patients with AKI would have a lower in-hospital mortality rate (Hassan et al., 2021). The association between race and AKI in PJI remains unknown, but warrants further investigation to improve prognosis.

Previous studies reporting AKI after revision surgery for aseptic reasons were scarce. Yadav A et al (Yadav et al., 2018). evaluated 2147 patients using to the RIFLE criteria and found the incidence of AKI to be 3.4%, which is similar to the incidence of primary TJA. The present evidence further revealed the hazardous nature of PJI, and its associated renal burden should be of additional concern.

### 4.2 Associated factors of AKI

The natural question for surgeons is: “What can be modified intraoperatively to reduce the development of AKI?” Our results revealed that hypoalbuminemia was an independent predictor of AKI following the first-stage exchange (OR= 3.593; *P*= 0.001). To our knowledge, this is the first study to assess the association between hypoalbuminemia and AKI in the field of arthroplasty. One previous study showed that hypoalbuminemia rather than systemic inflammatory response syndrome was a predictor of AKI in the intensive care unit (ICU) setting (OR=2.17) (Chawla et al., 2005). Another meta-analysis including surgical and ICU patients demonstrated that hypoalbuminemia was an independent risk factor for AKI (OR= 2.34) and AKI-related death (OR= 2.47) (Wiedermann et al., 2010). Our results further suggested that routine preoperative measurement of serum albumin may help to identify PJI patients with a higher risk of developing AKI. The next key question is whether this potential association may provide a rationale for changes in clinical management. As a surrogate indicator of malnutrition, hypoalbuminemia has been proven to be strongly associated with failure of a two-stage exchange protocol (Green et al., 2023). Some factors may confound this proposed association, such as a high systemic inflammatory state, poor lifestyle habits such as smoking, and chronic wasting disease, which may accompany hypoalbuminemia. The present study did not evaluate the effect of these factors on hypoalbuminemia (Poston and Koyner, 2019; An et al., 2021; Li et al., 2022). However, the current data could give us more sufficient evidence to help patients restore normal serum albumin levels preoperatively. However, this issue will only be better addressed if serum albumin causally improves clinical outcomes rather than acting as a simple marker for pathological processes.

The renoprotective effect of albumin was mediated by scavenging reactive oxygen species, preventing oxidative damage, and binding and delivering protective lysophosphatidic acid (Wiedermann et al., 2010). In a recent study, Angerett NR et al (Angerett et al., 2022). reduced the incidence of AKI after TJA from 6.71% to 4.15% by implementing a perioperative renal protocol. The present study may offer a new perspective to improve this protocol by correcting perioperative serum albumin levels.

Advanced age was associated with a higher risk of AKI in our cohort, which is consistent with previous results (Yadav et al., 2018; Theil et al., 2021). Similarly, a recent retrospective study of 390,382 patients showed a progressive increase in the incidence of postoperative AKI with age (Privratsky et al., 2023).

Additionally, a systematic review revealed that the presence of AKI was multifactorial, with acute renal blood flow impairment, poorer baseline renal function, and the use of nephrotoxic drugs as potentially important factors. Hypovolemia and acute renal blood flow impairment could lead to insufficient renal blood perfusion, resulting in prerenal acute kidney injury. Besides, perioperative improvement of patient blood volume to protect the kidneys is now actively advocated (Goren and Matot, 2015). These results are understandable and underline the importance of improving renal perfusion and restricting nephrotoxic drug regimens in high-risk patients. Future exploration of the availability of nephroprotective protocols in PJI patients is warranted. Notably, despite considerable effort, only a few studies have confirmed the association of type or dose of antibiotics with AKI, while more studies have not proven such an association. A noteworthy example was the large single-center study by Dagneaux L et al, which found that the risk of postoperative AKI in the TKA revision cohort was associated with increased vancomycin or aminoglycoside concentrations in the spacer (Dagneaux et al., 2021a), whereas AKI after THA septic revision could not be attributed to the type, dose or concentration of antibiotics given in the spacer or intravenously (Dagneaux et al., 2021b). The rationale for this phenomenon was still not clear. Possible reasons were as follows: firstly, there was a high degree of heterogeneity between studies, as the type and dose of antibiotics were often adjustable, resulting in differentiated results; secondly, these data indicated that the pathogenesis of AKI following first-stage exchange was multifactorial, with host-related factors likely to play an even more critical role, whereas the available evidence suggested that the administration of nephrotoxic antibiotics within reasonable doses (less than 8 g/40 g cement (Li et al., 2023)) may not significantly increase the risk of AKI.

### 4.3 Renal outcomes

The incidence of AKI requiring acute dialysis following first-stage exchange reported in the literature varies widely. The results of our cohort were similar to those of Dagneaux L et al (Dagneaux et al., 2021a; Dagneaux et al., 2021b), indicating that almost no patients required dialysis during hospitalization. However, another study reported a 3.7% dialysis requirement rate (Theil et al., 2021). This variation may be caused by the different indications for dialysis across institutions. Long-term renal outcomes after AKI remain unclear. In our cohort, 20% of AKI patients without pre-existing renal disease developed CKD after 90 days. Previous studies have reported progression to CKD in approximately 2-4% of patients with normal renal function, accounting for 15%-60% of patients with AKI (Dagneaux et al., 2021a; Dagneaux et al., 2021b). These findings should be interpreted with caution as relevant data are scarce.

### 4.4 Limitations

Several limitations in present study are worth mentioning. Firstly, the design was retrospective, and certain biases of retrospective study are inherent. Secondly, limited by the unavailability of data, the impact of perioperative fluid management, antibiotic dose, and anesthetic factors on AKI could not be assessed in our cohort. However, as an updated systematic review of the current topic, we summarized the present research evidence and illustrated the multifactorial nature of AKI pathogenesis. One point of interest was the association between antibiotic dose and AKI. We found only a few publications supporting that a higher antibiotic dose in two-stage revision may lead to the development of AKI, however, we were not able to further evaluate the relationship between antibiotic dose and AKI in our cohort. Future, more thorough studies could investigate this issue. Thirdly, the studies included in the meta-analysis were heterogeneous in terms of patient characteristics, surgical technique, and AKI diagnosis. Future studies based on large-scale databases are still needed. Fourthly, the incidence of AKI is probably underestimated, as information about novel AKI biomarkers and urine volume were lacking and some patients may be discharged before they meet the AKI criteria. Approximately 10% of patients in our cohort were discharged within 7 days postoperatively, but their AKI prevalence was not significantly reduced (4/34). Furthermore, although we found an association between preoperative hypoalbuminemia and AKI, this conclusion is still preliminary, and future prospective studies with close renal monitoring are warranted. However, given that this association has been demonstrated in a variety of other diseases, clinicians should be vigilant in such patients.

## 5 Conclusions

In conclusion, a meta-analysis including 2525 PJI patients indicated the incidence of AKI following first-stage exchange was 16.6%. Current evidence suggested that the pathogenesis of AKI is multifactorial, including host characteristics, factors contributing to acute renal blood flow injury, and the use of nephrotoxic drugs. Hypoalbuminemia may be a novel factor associated with AKI development. Although the need for acute dialysis in the case of AKI is uncommon, the fact that some AKI patients will develop CKD still needs to be taken into consideration. Further high-quality studies are needed to investigate approaches to improve the occurrence and progression of AKI.

## Data availability statement

The original contributions presented in the study are included in the article/**Supplementary Material**. Further inquiries can be directed to the corresponding author.

## Ethics statement

The studies involving humans were approved by Chinese PLA General Hospital Ethics Committee. The studies were conducted in accordance with the local legislation and institutional requirements. The ethics committee/institutional review board waived the requirement of written informed consent for participation from the participants or the participants’ legal guardians/next of kin because retrospective study, which was approved by the Ethics Committee.

## Author contributions

ZL provided idea. ZM, ZL, JF, and L-BH analyzed the data and performed the experiments. ZL, ZM, and FY wrote the manuscript. CX and J-YC assisted in revising the manuscript. CX and J-YC gave important guidance and analysis in the review process. All authors contributed to the article and approved the submitted version.
