# Supplementary material for: Incidence, associated factors, and outcomes of acute kidney injury following placement of antibiotic bone cement spacers in two-stage exchange for periprosthetic joint infection: a comprehensive study
Source: Front Cell Infect Microbiol. 2023 Sep 20;13:1243290. doi: 10.3389/fcimb.2023.1243290 (PMC10548219; doi:10.3389/fcimb.2023.1243290)

Supplementary Material

**Supplementary Table 1**. Search terms for systematic review.

| **Pubmed** | ((((((((((acute kidney injury) OR renal failure) OR AKI) OR kidney failure) OR renal insufficiency) OR dialysis) OR hemodialysis) OR hemofiltration) OR renal function) OR nephrotoxicity)) AND ((((((((periprosthetic joint infection) OR PJI) OR prosthetic Joint Infection) OR septic loosening) OR aseptic loosening) OR prosthesis-related infections OR spacer OR revision)  Total: 955 |
| --- | --- |
| **MEDLINE** | #1  'periprosthetic joint infection'/exp  #2  'prosthetic joint infection'/exp  #3  'septic loosening'/exp  #4  'prosthesis infection'/exp  #5  'spacer'/exp  #6  'revision arthroplasty'/exp  #7  #1 OR #2 OR #3 OR #4 OR #5 OR #6  #8  'kidney failure'/exp  #9  'kidney injury'/exp  #10  'dialysis'/exp  #11  #8 OR #9 OR #10  #12  #7 AND #11  Total: 612 |
| **Cochrane** | Search all text "acute kidney injury" AND "periprosthetic joint infection" |

**Supplementary Figure 1**. Funnel plot evaluating for publication bias evaluating incidence of AKI in patients undergoing first-stage exchange.


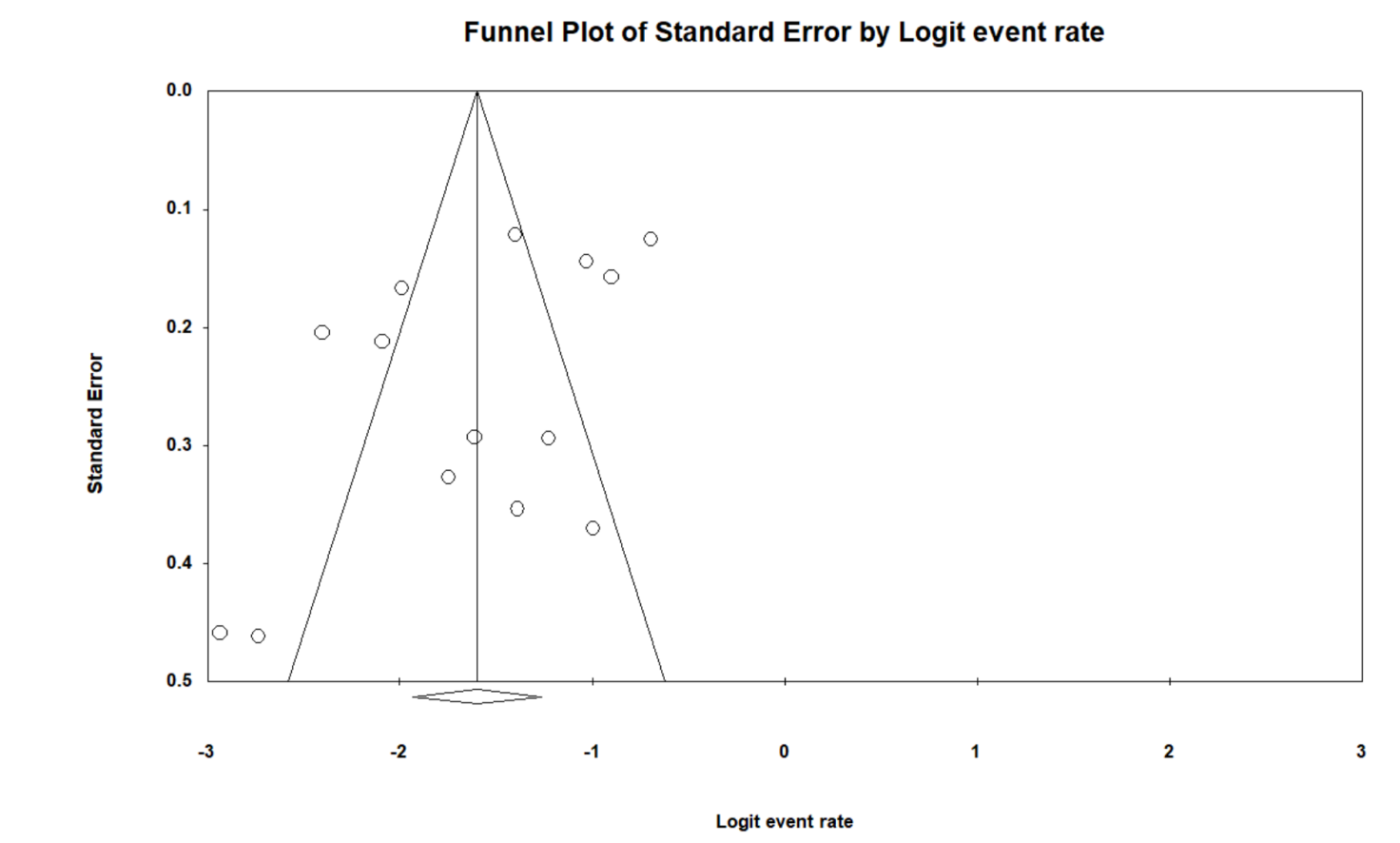

Supplement: Supplementary file 1 [file DataSheet_1.docx]
